# Does the type of anesthesia (regional vs. general) represent an independent predictor for in-hospital complications in operatively treated malleolar fractures? A retrospective analysis of 5262 patients

**DOI:** 10.1007/s00068-023-02235-7

**Published:** 2023-02-15

**Authors:** Claudio Canal, Alexander Kaserer, Laurent Sébastien Morax, Franziska Ziegenhain, Hans-Christoph Pape, Valentin Neuhaus

**Affiliations:** 1 Klinik für Traumatologie, Universitätsspital Zürich (USZ), Universität Zürich (UZH), Rämistrasse 100, 8091 Zurich, Switzerland; 2 Klinik für Anästhesie, Universitätsspital Zürich (USZ), Universität Zürich (UZH), Rämistrasse 100, 8091 Zurich, Switzerland; 3 Klinik für Anästhesie, Kantonsspital Luzern (LUKS), Spitalstrasse 16, 6000 Lucerne, Switzerland

**Keywords:** Malleolar fracture, Complications, Anesthesia

## Abstract

**Purpose:**

The impact of the type of anesthesia (regional vs. general anesthesia) on in-hospital complications in ankle fractures has not been thoroughly studied yet. Identifying factors that place patients at risk for complications following ankle fractures may help reduce their occurrence. The primary goal of this study was (1) to describe the cohort of patients and (2) to evaluate independent risk factors for complications during hospitalization.

**Methods:**

We analyzed patients from 2005 to 2019 with an operatively treated isolated fracture of the medial or lateral malleolus using a prospective national quality measurement database. Patients were selected based on international classifications (ICD) and national procedural codes (CHOP). Uni- and multivariate analysis were applied.

**Results:**

In total, we analyzed 5262 patients who suffered a fracture of the malleolus; 3003 patients (57%) had regional and 2259 (43%) general anesthesia. Patients with regional anesthesia were significantly older (51 vs. 46 years), but healthier (23 vs. 28% comorbidities) than patients who received general anesthesia. The in-hospital complication rate was not significantly lower in regional anesthesia (2.2% vs 3.0%). The type of anesthesia was not an independent predictor for complications while controlling for confounders.

**Conclusion:**

Type of anesthesia was not an independent predictor of complications; however, higher ASA class, age over 70 years, fracture of the medial versus lateral malleolus, longer preoperative stay, and duration of surgery were significant predictors of complications. Patient and procedure characteristics, as well as changes in medical care and epidemiological changes along with patient requests, influenced the choice of the type of anesthesia.

## Introduction

Ankle fractures are common and often require hospitalization [1]. They typically occur in young and active patients or older patients with reduced bone quality [2]. Previous studies found an increase in the incidence and severity of ankle fractures in recent years, mainly due to the aging population [3–5]. In a large Swedish registry study, over 80% of ankle fractures required operative treatment [6]. Depending on the patients age and comorbidities, different types of anesthesia may be preferred. Surgical treatment of ankle fractures is usually performed under general or regional anesthesia. Previous research on hip and knee arthroplasty suggests regional anesthesia may have lower complication rates and shorter hospital stays compared to general anesthesia [7–10].

However, the influence of the type of anesthesia on short-term complications in ankle fractures has not been well studied**.** Identifying factors that place patients at risk for complications following ankle fractures may help to reduce their occurrence.

This study aimed to investigate the association between the type of anesthesia and in-hospital complications in patients with operatively treated ankle fractures.

## Methods

### Study design

This study was conducted using a prospective surgical registry from the “Working Group for Quality Assurance in Surgery” (known in German as “Arbeitsgemeinschaft für Qualitätssicherung in der Chirurgie”, AQC). The goal of the AQC is to collect data on various operative interventions and surgical diseases and injuries [11]. The aim is to ensure quality assurance in surgery. Over 80 Swiss surgical departments register their surgical in-hospital cases online using the AdjumedCollect tool [12].

The AQC database is based on a two-part questionnaire. The first part contains information concerning the operation(s). Patient and general hospitalization information is collected in the second part of the questionnaire.

The AQC data include information on intraoperative and postoperative complications as well as general case-related complications. These are classified according to complications in the context of surgical access, positioning of the patient, the wound, and the corresponding operation performed. The case-related complications are listed by organ system, e.g., pulmonary, cardiac, and gastrointestinal with the most relevant respective associated diagnoses. Pain or a necessary reoperation was not counted as a complication per se, but the cause for which a reoperation became necessary, e.g., rotation error or instability.

The study has been approved by the institutional review board—no approval of the local cantonal ethical review board was needed because no identifiable patient information is registered in the AQC database. The present study was performed according to the Strengthening the Reporting of Observational Studies in Epidemiology (STROBE) guidelines [13].

### Study subjects

All patients found in the AQC register between January 1, 2005, and December 31, 2019, with an operatively treated isolated fracture of the medial or lateral malleolus were included in our study. We identified patients with the corresponding World Health Organization (WHO) International Classification of Disease Code (ICD-10) [14] S82.5 (fractures of the medial malleolus) and S82.6 (fractures of the lateral malleolus).

Exclusion criteria included a lack of information on the operation or the patient.

From 5839 cases, a total of 5262 patients met our criteria and were included for further examination.

### Variables and outcome measures

The aim of this retrospective study was to investigate the outcome differences in malleolar fractures depending on the type of anesthesia used.

Possible confounders, such as age, sex, ASA classification, type of injury, experience level of surgeon, surgery and ICU duration, thromboembolism and antibiotic prophylaxis were assessed.

### Statistical analysis

Analysis between groups of categorical data was done using the Chi-squared test and presented as the number of patients and percentages. The Student’s *t*-test was used to assess differences in means between groups for numerical data.

Binary logistic regression was used to determine independent risk factors for complications. A *p* < 0.05 was considered statistically significant. SPSS Version 26 (IBM, Armonk, New York, USA) was used to analyze the data.

## Results

This study included a total of 5262 patients who suffered a fracture of the medial or lateral malleolus with a mean age of 49 years. Fifty-one percent of the patients were male.

In total, 57% (*n* = 3003) of the patients received a regional anesthesia. Fractures of the lateral malleolus were proportionally more frequently operated under regional anesthesia.

Patients with regional anesthesia were significantly older (51 vs. 46 years), healthier (lower ASA-classification), with less comorbidities (23% vs. 28%) than patients who received general anesthesia (*p* < 0.001) (Table 1).

**Table 1:** Type of anesthesia; Patient characteristics

Parameter	General anesthesia (*n* = 2259)		Regional anesthesia (*n* = 3003)		p value
	*n*	%	*n*	%	
*Age (years)*					
Mean ± SD	46 ± 19		51 ± 17		< 0.001
*Sex*					
female	1052	46.6	1507	50.2	0.009
Male	1207	53.4	1496	49.8	
*ASA*					
I (healthy person)	1199	53.1	1749	58.2	< 0.001
II (mild systemic disease)	928	41.1	1150	38.3	
III (severe systemic disease)	132	5.8	104	3.50	
*Insurance*					
Statutory	1680	74.4	2188	72.9	0.220
Private	579	25.6	815	27.1	
*Diagnosis*					
S82.5 fracture of medial malleolus	317	14.0	319	10.6	< 0.001
S82.6 fracture of lateral malleolus	1942	86.0	2684	89.4	
*Length of stay (days)*					
Median and (IQR)	7.0 (6)		5.0 (6)		< 0.001
*Length of stay preoperative (days)*					
Median and (IQR)	2.0 (4)		1.0 (3)		< 0.001
*Length of stay postoperative (days)*					
Median and (IQR)	4.0 (4)		4.0 (3)		< 0.001
*Need for intensive care*					
Yes	33	1.5	14	0.46	< 0.001
*Comorbidity*					
Yes	642	28.4	692	23.0	< 0.001
*Complications during hospital stay*					
Yes	67	3.0	66	2.2	0.079
*Discharge*					
Deceased	4	0.18	11	0.37	0.023
At home	2099	92.9	2844	94.7	
Rehabilitation clinic	90	4.0	79	2.6	
Nursing home	23	1.0	24	0.80	
Old people's home	24	1.1	19	0.63	
Other hospital	19	0.84	26	0.87	

*SD* standard deviation, *ASA* American Society of Anesthesiologists classification system, *n.s.* not significant, *(IQR)* interquartile range

Patients with regional anesthesia were operated on by more experienced surgeons (*p* < 0.001) and had shorter procedural duration (*p* < 0.001). In addition, the regional anesthesia cohort received fewer prophylactic antibiotics (*p* < 0.001). Table 2).

**Table 2:** Type of anesthesia; procedure characteristics

Parameter	General anesthesia (*n* = 2259)		Regional anesthesia (*n* = 3003)		*p* value
	*n*	%	*n*	%	
*Surgeon class*					
Senior attending	697	30.9	1224	40.8	< 0.001
Junior attending	882	39.0	1055	35.1	
Resident	680	30.1	724	24.1	
*Duration surgery (minutes)*					
Mean ± SD	68 ± 36		62 ± 29		< 0.001
*Thromboembolism prophylaxis during hospital stay*					
No thromboembolism prophylaxis	81	3.6	89	3.0	0.449
Thromboembolism prophylaxis	2101	93.0	2810	93.6	
Anticoagulation	77	3.4	104	3.5	
*Antibiotics during hospital stay*					
No antibiotics	170	7.5	335	11.2	< 0.001
Prophylactic antibiotics (before start of surgery)	2021	89.5	2603	86.7	
Prophylactic antibiotics (after start of surgery)	36	1.6	39	1.3	
Antibiotic therapy	32	1.4	26	0.87	
*Operation*					
Open reduction, internal fixation	1997	88.4	2760	91.9	< 0.001
Closed reduction, internal fixation	99	4.4	115	3.8	
External fixator	104	4.6	73	2.4	
Internal fixation	20	0.89	22	0.73	
Open reduction of a luxation	18	0.80	17	0.57	
Closed reduction without internal fixation	11	0.49	10	0.33	
Closed reduction of a luxation	10	0.44	6	0.20	

*SD* standard deviation

**Table 3:**
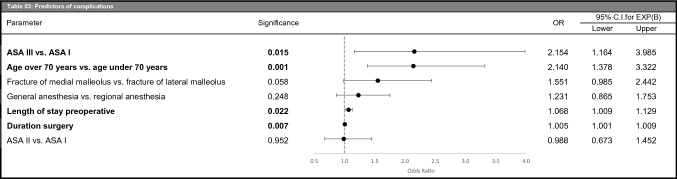
Predictors of complications

ASA: American Society of Anesthesiologists classification system, OR: odds ratio

### Outcome

Patients with regional anesthesia had a statistically significant shorter length of stay (6.7 vs. 7.8 days; *p* = < 0.001). Overall complications were comparable between both groups (3.0% vs. 2.2%; *p* = 0.079). The mortality rate did not differ, neither comparing the main diagnosis nor the type of anesthesia.

The most common reported intraoperative complications were lesions of the artery or tendon, a peripheral nerve lesion, thermal lesion, and iatrogenic fracture. The most common postoperative complications reported were wound-healing disorder, wound infection, secondary dislocation, and instability. The most common reported case-related complications were myocardial infarction, respiratory insufficiency, urinary tract infection and acute psychosis.

In multivariate analysis for any complications, the anesthesia type was not an independent risk factor. But a higher ASA class (III versus I), age over 70 years, fracture of medial versus the lateral malleolus, a longer length of stay preoperative, and duration of surgery were independent risk factors for complications (Table 3).

## Discussion

This study aimed to evaluate independent risk factors for complications during hospitalization for patients with operatively treated malleolar fractures, with a focus on the type of anesthesia used. To the best of our knowledge, this has not been studied well. Identifying factors that place patients at risk for complications following ankle fractures may help to reduce their occurrence.

There are different types of anesthesia for fixation of malleolar fractures, mainly regional or general anesthesia [15]. The choice of anesthesia may affect peri-operative recovery and pain control [16], which can impact patient satisfaction with their treatment [17].

In our study, patients with regional anesthesia were significantly older, but healthier than patients who received general anesthesia. Anesthesia type was not an independent predictor for complications after controlling for confounders. This main finding aligns with previous research [18, 19] who showed no differences in complications and mortality comparing spinal and general anesthesia in ankle fractures.

A higher ASA-Score, the age over 70 years, the length of stay preoperative and the duration of surgery were significant predictors for complications in our study.

The importance of the ASA-class on complications in ankle fractures has been previously described [20, 21], especially on surgical site infection [22].

We also found the often-described bimodal age distribution in our cohort [5, 23]. There exist significant differences between men and women regarding age, lifestyle, comorbidities, and the type of ankle fracture sustained [24]. The complication rate in surgically treated ankle fractures increases with age [20, 21, 25]. Danilkowicz et al. found a linear increase in complications after open reduction and internal fixation of ankle fractures with age in 27,633 patients [25]. Interestingly, Gil et al. showed that patients aged 80–89 had a complication rate similar to patients aged 65–79 after controlling for the ASA class [26].

A longer length of stay preoperative is associated with poorer clinical outcomes [27] and poorer quality of life of patients [28, 29]. This relationship may be due to occurred complications who need more time to treat and other factors.

Contrary to the findings of our study, Vora et al. reported that patients with spinal anesthesia for ankle ORIF had a longer length of stay in the hospital [18].

In contrast, we found that the group with regional anesthesia had a shorter length of stay.

Similar to our study, other researchers found that the duration of surgery is an independent risk factor for complications [21, 30]. Louie et al. found that resident involvement per se is not a significant risk factor in malleolar fractures [21].

The effect of our two continuous measures (length of stay and duration of surgery) seems to be rather small. This is because the odds ratio shown only shows the change per additional unit (one day or one minute).

In the past 20 years, there has been a global increase in obesity [31]. A higher bodyweight increases the risk of experiencing a severe ankle fracture in a linear fashion [32]. Obese patients tend to have worse long-term outcomes following a fracture. In our study, we did not have enough data on patient weight to further investigate this effect or other medical conditions that may influence the outcome or more details in fracture type, which also play a role in the outcome [33, 34].

As expected for isolated extremity fractures, the mortality rate in our study was very low,

which is consistent with the findings of previous research on this topic [35, 36].

It is expected that in coming years, older, and sicker patients will need surgery for ankle fractures [37]. This, along with socioeconomic changes, may influence the choice of anesthesia. This trend has already been partially noticed in patients undergoing hip fracture surgery [38]. Patient’s characteristics and the type of surgery may influence the choice between general and regional anesthesia [39].

One potential consideration when choosing the type of anesthesia is the ease of postoperative recovery and the effectiveness of pain control [16]. These factors may impact overall patient satisfaction with their treatment [17].

## Limitation

A strength of our study is the large sample size. Because of the nature of the data source, our study has various limitations. One important limitation is the use of de-identified data, which made it impossible to obtain missing information. Furthermore, we only had information about the course of events during hospitalization and no data about further course. Another weakness, as in many registry studies, is the quality of collecting and recording data, which is dependent on many different physicians.

The findings of our study may not be generalizable in other populations or settings, as the study is based on the specific situation and medical procedures in Switzerland.

The results of this study may be affected over time by changes in practices or technologies, which may affect the findings. Especially the development of new drugs and techniques has greatly enhanced the safety of anesthesia, reducing the risk of complications and side effects [40]. In addition, we did not have more detailed information about the anesthesia such as who performed the anesthesia and what technique, and equipment was used.

## Conclusion

Type of anesthesia was not an independent predictor of complications; however, higher ASA class, age over 70 years, fracture of the medial versus lateral malleolus, longer preoperative stay, and duration of surgery were significant predictors of complications. Patient and procedure characteristics, as well as changes in medical care and epidemiological changes along with patient requests, influenced the choice of the type of anesthesia.

## Data Availability

The data that support the findings of this study are available from the corresponding author, [Claudio Canal], upon reasonable request.
